# Challenges in Diagnosing Occupational Chronic Obstructive Pulmonary Disease

**DOI:** 10.3390/medicina57090911

**Published:** 2021-08-30

**Authors:** Cristiana Libu, Marina Ruxandra Otelea, Ioan Anton Arghir, Agripina Rascu, Sabina Antonela Antoniu, Oana Cristina Arghir

**Affiliations:** 1 Department of Pneumology, Faculty of Medicine, Ovidius University of Constanta, 900470 Constanta, Romania; libu.cristiana@yahoo.com (C.L.); ionut_arghir93@yahoo.com (I.A.A.); oana.arghir@univ-ovidius.ro (O.C.A.); 2 Clinical Department 5, Faculty of General Medicine, University of Medicine and Pharmacy Carol Davila, 020021 Bucharest, Romania; agripina.rascu@umfcd.ro; 3 Department of Occupational Medicine, Colentina Clinical Hospital, 020125 Bucharest, Romania; 4 Department of Medicine II and Palliative Care Nursing, University of Medicine and Pharmacy Grigore T Popa, 700115 Iasi, Romania; sabina.antoniu@umfiasi.ro

**Keywords:** COPD-exposure hazards, inhaled noxious compounds, occupational medicine, workplace

## Abstract

Occupational chronic obstructive pulmonary disease (oCOPD) represents 15–20% of the global burden of this disease. Even if industrial bronchitis has long been known, new occupational hazards continue to emerge and enlarge the number of people exposed to risk. This review discusses the challenges related to the early detection of oCOPD, in the context of new exposures and of limited usage of methods for an efficient disease occupational screening. It underlines that a better translation into clinical practice of the new methods for lung function impairment measurements, imaging techniques, or the use of serum or exhaled breath inflammation biomarkers could add significant value in the early detection of oCOPD. Such an approach would increase the chance to stop exposure at an earlier moment and to prevent or at least slow down the further deterioration of the lung function as a result of exposure to occupational (inhaled) hazards.

## 1. Introduction

The estimated prevalence of chronic obstructive pulmonary disease (COPD) worldwide is 13.1%, with variations between 11.6 and 13.9% among different global regions [1]. COPD is also the leading cause of mortality among deaths related to respiratory diseases and the third leading cause of overall mortality at the global level [2,3]. These two indicators are significant for the importance of COPD as a public health problem throughout the world and are imperative for identifying effective prevention and treatment measures.

It is undeniable that COPD prevalence is closely related to that of smoking, which is recognized as the main etiologic factor for disease development in 55–75% of the cases [4]. Despite the declining trend in COPD prevalence with reduced smoking exposure [5,6,7], the number of COPD cases remains high and, therefore, requires new prevention strategies [8]. One of the explanations behind this behavior lies in the etiological role of other risk factors and in their increasing impact. Thus, the low economic level, which largely overlaps with occupational exposure to respiratory hazards, has significantly increased the prevalence of COPD, even in economically developed countries, where workers’ means of protection are well established and relatively effective [9].

More recent estimations consider that 15–20% of COPD cases are due to occupational exposure [10]. The etiological fraction of oCOPD was 19% in smokers and 31% in non-smokers, after adjustments made according to sex, age, smoking status and socio-economic status [11]. These findings underly the importance of early detection of the relationship between the disease and occupational exposure [12]. Early recognition of occupational etiology is essential because, ideally, once a risk factor of COPD has been identified in the workplace, exposure must be discontinued, or if this is not possible, at least diminished.

Unfortunately, at this time, effective prevention has at least two critical barriers. The first barrier consists in defining COPD, which involves recognizing this entity in a late phase of evolution, in which bronchial obstruction is irreversible. Occupational exposure cessation and treatment could be more effective in the early stages of COPD. The second barrier is a characteristic of all occupational diseases. It refers to the profound social implications of changing jobs before evolving to an advanced stage of the illness or to repeated exacerbations. Continued occupational exposure maintains the pathophysiological vicious circle and reduces the effectiveness of therapeutic solutions.

Therefore, this review is dedicated to presenting the current challenges in oCOPD and possible ways to overcome them.

## 2. Etiology of oCOPD: New Workplace Determinants, New Challenges

Industrial bronchitis has long been known since 1966 [13]. Except for smoking, occupational exposure to respiratory risk factors is generally higher than non-occupational exposure (both in intensity and duration). Therefore, as in other chronic occupational pathologies, the age of onset is lower than the occurrence of the disease, in which there is no occupational determinism. Thus, the prevalence of oCOPD in people exposed to gases, fumes and vapors is two times higher in the range of age <40 years [8]. In this early age interval, except for pathologies with a significant genetic component, such as alpha-1 antitrypsin deficiency, non-occupational COPD is relatively rare. However, the difference in prevalence is also maintained for the age range of 40–60 years [8]. This fact is consistent with the more general findings that the COPD phenotype in non-smokers occurs at younger ages in women, especially if there is a tendency to be overweight [14]. Lung function decline is less abrupt, emphysema is less common, but small airways dysfunction is more common and sputum is more often eosinophilic asthma-like [15]. However, unlike asthma, there is no improvement with corticosteroids treatment [15].

In classical occupational pathology, the chronic bronchitis phenotype tends to be better expressed than the emphysematous one, except for the exposure to cadmium [16,17] and coal dust [18]. In all these cases, the centrilobular type of emphysema is described [19].

The occupations with the highest incidence of COPD are the mining, textile, agriculture, machine-building and chemical industries [20,21,22,23,24]. The most common causes of occupational chronic bronchitis are presented in Table 1.

Several meta-analyses published in recent years have highlighted an increased risk for oCOPD in people exposed to fumes and welding gases, pesticides, inorganic, or organic dust [25,26,27]. A large cohort study, which included over 8000 subjects, followed up for 20 years, showed a 70% increased risk of oCOPD in people exposed to metals, while the risk increase was 72% in people exposed to mineral dust [28]. Welders are routinely exposed to vapors, gases, dust and fumes (VGDF); numerous studies published in recent years have identified these occupational risk factors in patients with COPD [29]. Thus, an analysis performed on 2736 subjects with known occupational exposure investigated by quantitative computed tomography of the lung showed that 49.9% of them had a history of present or past exposure to VGDF [30]. Another study identified a different phenotype of COPD, secondary to VGDF exposure, in which the frequency of wheezing and allergic rhinitis is higher than in other COPD patients [31]. In the same study, for a similar level of airflow limitation, patients with VDGF exposure had a current and cumulative level of smoking exposure lower than subjects with COPD who were never exposed to occupational hazards. Moreover, the association with VDGF exposure is perfectly explicable because this type of occupational exposure shares multiple pathogenic similarities with the tobacco smoking exposure. Furthermore, alike environmental exposure, VDGF exposure is a recognized cause of lung cancer in never smokers in a comparable manner for smoking exposed counterparts [32,33]. Rarely, authors described COPD in workers exposed to metal-working fluids, arsenic, indium, manganese, gases emitted by diesel engines used in transportation or construction, cleaning substances, or cellulose production factories [34,35,36,37,38,39,40].

Postmodern society has extended the understanding of oCOPD etiopathogenesis to categories previously not considered to be at risk of significant occupational hazards, by assuming and then confirming the relationship between workplace exposure and disease. Some of the occupations are even a “surprise” for the statistics: for example, the Center for Disease Control and Prevention (CDC) showed that the highest number of oCOPD cases was among workers of the computer industry (3.3%) as well as for office and administrative professionals (3.3%) [41]. Prolonged exposure to ozone, nitrogen or sulfur dioxide, or ammonium, initially described as sick building syndrome could be the explanation, at least in part for these oCOPD [42]. White-collar workers who are routinely in contact with submicrometer particle emissions released by copiers are more likely to develop a cough with sputum [43]. Theoretically, air conditioning systems help to ensure a comfortable temperature, which could be a protective factor. However, supposing the air conditioning systems have malfunctioned, the effect is the opposite, namely a reduction in ventilation lung function with chronic bronchitis, independent of the incidence of hypersensitivity pneumonitis [44,45,46].

A new occupation at risk for oCOPD is cooking. A study conducted in Norway confirmed an increased risk in those exposed to smoking from food preparation, especially smoke from burning wood and coal in grills [47].

As the recycling industry of some materials develops, the cases of bronchial obstruction diagnosed in these people are related to the inflammation generated by exposure to bioaerosols, volatile chemicals, metals and infectious agents, endotoxins, β (1–3)-glucans, mycotoxins [48]. In addition to experimental data, clinical studies have shown that people who collect and store solid waste have acquired a significant reduction in forced expiratory volume in 1 s (FEV1) and Tiffneau index [49,50,51]. A relatively recent risk factor is represented by the exposure to biomass in the industrial production of energy.

Industrial exposure to wood aches, endotoxins, volatile organic compounds for example, in large-scale biomass combustion plants is also recognized as a workplace hazard for oCOPD [52]. Furthermore, its pathogenic consequences seem to be more significant than those of smoking exposure. For the same level of FEV1 decline, biomass-induced COPD was associated with a higher risk of impaired gas exchange and higher level of oxygen desaturation.

The challenge for the occupational physician is to investigate and report the new respiratory hazards, because only from a close monitoring of the workers [53] might further public health actions be proposed.

## 3. oCOPD: Diagnostic Challenges

Early recognition of the disease remains the main challenge for the physician. Occupational COPD, once diagnosed, follows the same treatment regimens and pulmonary rehabilitation as non-occupational COPD, the only specific recommendation being to discontinue occupational exposure.

An estimation of exposure is possible with proper occupational history. This should include questions about the career path, the exposure and the exposure–symptoms timeline relationship. In most cases of occupational chronic bronchitis, recognizing respiratory irritants as etiological agents, the presence of cough, accompanied or not by dyspnea, rhinorrhea and/or eye irritation during the occupational activity may be suggestive enough for a work-related disease.

There are many possibilities to improve the current medical surveillance of subjects exposed to respiratory hazards. Currently, this is achieved by spirometry, a functional investigation that does not detect COPD at an early stage. Therefore, other screenings and diagnosis methods have been proposed. These methods aim at three directions: better investigation of the respiratory physiology, improvement of the imaging diagnosis and usage of biomarkers to monitor inflammation. A faster translation of these methods into clinical practice could add significant value in the early detection of oCOPD.

### 3.1. Airflow Limitation—A Functional Hallmark of oCOPD

The functional pattern in COPD is obstruction associated with hyperinflation, changes in gases transfer and blood gases.

According to the Global Initiative for Chronic Obstructive Lung Disease (GOLD), the diagnosis and severity of COPD is evaluated by spirometry. Although COPD accounts for numerous and quite heterogeneous pathophysiological abnormalities, staging of COPD includes only spirometry, as the gold standard method for the airflow obstruction and the modification of FEV1 after bronchodilation. However, spirometry remains an effort-dependent technique and a complex procedure to be performed. Factors causing airflow limitation also increase airway resistance, traditionally measured by body plethysmography parameters as specific airway resistance (sRaw) and conductance (sGaw). Plethysmography is considered extremely sensitive for airflow obstruction in COPD patients. Still, it is less commonly used than spirometry because it is more expensive than spirometry [54].

The forced oscillation technique (FOT) allows the determination of two primary components: respiratory system resistance (RRS) and reactance (XRS). On one side, FOT is much easier to perform than spirometry. On the other side, plethysmography (FOT) provides a more sensitive evaluation of airway obstruction than spirometry, which requires a deep inhalation, diminishing the airway resistance, at least in the initial stages of COPD [55,56].

Impulse oscillometry (IOS) is a specific type of FOT using rectangular waveform pulses instead of noise signals. IOS records the respiratory resistance at 5 and 20 Hz (R5 and R20, respectively), the reactance at 5 Hz (X5) and the resonant frequency (Fres) [55]. Reactance is considered more valuable than resistance measurements in revealing the changes in pulmonary mechanism induced by airflow obstruction in COPD [55]. Still, it is not clear which parameter of IOS is the most informative in assessing COPD’s severity [57].

Some authors consider that IOS might be a more sensitive technique in detecting the early-stage COPD and lung damage in COPD. The difference between R5 and R20 resistance and the area under the reactance curve correlate better than FEV1 with symptoms and exacerbations [58,59]. This is because FEV1 does not reflect the peripheral airways, whereas reactance (X5) can fill this gap and provide information about the changes in pulmonary compliance induced by airflow obstruction [60]. Frantz et al. also reported in subjects with COPD suggestive symptoms and normal spirometry, a higher pulmonary resistance and lower pulmonary reactance, supporting the idea that IOS is a more sensitive method in detecting the early subtle changes in lung function [61]. Several IOS studies considered an increase of R5 at low frequencies as representing the peripheral airway obstruction [62,63].

IOS could divide the COPD patients into four phenotypes: normal IOS group, resistance-dominant group, reactance-dominant and mixed ones with different grades of dyspnea, lung function impairment and emphysematous lesions [64,65].

The European Respiratory Society Task Force Report highlighted, in 2003, the urgent need for further large-scale studies of the measurements made by IOS and, almost two decades after, technical standards for respiratory oscillometry were published [66].

Welders and first responders to WTC disaster are among the few cases of occupational exposure in which the oscillometry was already used [67,68].

We do believe that the recommendation of The European Respiratory Society Task Force should be extended to the workers exposed to respiratory hazards, in view of its capacity to detect earlier changes than spirometry and accessibility to perform in ambulatory settings. In workplaces at risk of oCOPD, IOS should help assess the actual efficacy of workplace intervention.

### 3.2. Imaging Diagnosis Underuse in oCOPD

The parameters of traditional spirometry, mainly FEV-1, forced vital capacity (FVC) and forced expiratory flow at 25–75% (FEF 25–75), underestimate the diagnosis of COPD in subjects with minimal respiratory symptoms or with structural changes, observed only on CT. Therefore, new indicators for an early diagnosis are crucial. Imaging techniques complement the functional picture by directly measuring areas of emphysema and the dimensions of the airways. In this respect, CT could become an early diagnosis tool in the future. COPD would need an easy-to-measure peripheral obstruction, as the micro-computed tomography (micro-CT) scans are capable to reveal the obstruction of the terminal and transitional bronchioles before emphysema [69,70].

Computed tomography (CT)—respectively, high-resolution CT (HRCT) or micro-CT (already used in many clinical trials)—are methods that provide valuable information on the presence and distribution of emphysema and the degree of impairment of the small airways, responsible for increased airway resistance. Micro-CT detects the obstruction of the peripheral airways which precedes the onset of emphysema, which can be considered a marker for the early diagnosis of COPD [70]. The main disadvantage of these methods is the high cost, but the information generated by CT might serve as a reference for validating other forms of disease detection.

As mentioned, spirometry has certain limitations in detecting small airway obstruction, predominantly affected in COPD. This creates a discrepancy between the functional changes noticeable on spirometry and the extent of destructive lesions related to pulmonary emphysema. Thus, a study led by Sanders showed many years ago that CT shows lesions of emphysema in 69% of smokers with minimal or even absent obstructive changes [71].

Moreover, in a cohort study that included smokers and non-smokers, Regan et al. found that approximately half of the smokers and former smokers without spirographic signs of obstruction had structural lung damage on CT [72]. In a longitudinal study, Young et al. identified a tissue-airway endotype in 70% of patients whose initial lesions were in the small airways. The other patients with COPD were classified as the “airway-tissue” endotype, in which the pathology begins at the central level [73].

In another cohort study which included 8307 participants—smokers and former smokers—Bhatt et al. correlated the structural changes visualized by volumetric CT in maximum inspiration (corresponding to total lung capacity) and maximum expiration (corresponding to functional residual capacity) [74]. The authors managed to identify the usefulness of new spirometry parameters associated with mild airflow obstruction and emphysema lesions identified at CT.

Imaging investigation can also provide information about another risk factor for COPD: the type of bronchial arborization [75]. This element is essential in the exposure to microparticles because it can facilitate their retention, prolonging harmful effects. The branch variation of the central bronchial tree has a genetic determinism. A sub-segmental accessory airway is quite common in COPD and is associated with higher severity of the disease, probably because subjects with this anatomical variant have a slightly shorter length of the central component of the bronchial tree. Identifying such anatomical variants and their genetic substrate could allow a correct stratification of the individual risk [75].

### 3.3. Integration of New Inflammatory Markers in Monitoring the Exposed Workers

Inflammation markers can be determined systemically (in the blood or urine) or locally (in the lavage fluid, sputum, or expired air). Studies specifically dedicated to occupational pathology are very few, but the data obtained from research conducted in the general population could translate to occupational pathology. For example, a study on 4697 people found that for every 10 μg/m^3^ increase in exposure to 2.5 μ particles (PM2.5 exposure), FVC, FEV1 and PEF are reduced by 2.94 mL/s, 2.02 mL/s and 16.14 mL/s, respectively. At the same time, researchers highlighted the role of 8-iso-PGF2α and 8-iso-PGF2α in mediating the relationship between exposure to microparticles and the decline of lung function [76].

The advantage of determining markers of inflammation (cytokines, leukotrienes) and oxidative stress factors (products of lipid peroxidation, nitric acid metabolism, hydrogen peroxide, or hydrogen ions) in the expired air provides valuable information about the pathogenesis of COPD. They are, at least theoretically, the ideal screening methods because they are non-invasive, collected by portable equipment, allowing repeated measurements in outpatients. As they are easily repeated, these methods could be used before and after the occupational exposure and indicate the differences. The measurement of the fraction of the exhaled nitric oxide (FeNO) has the advantage of being well standardized in asthmatics. By now, the results of studies performed on patients with COPD gave contradictory results [77]. A systematic review showed a tendency to higher FeNO values in COPD compared to controls but highlighted the high heterogeneity of the studies included in the meta-analysis [78]. The authors stratified data from studies that included COPD patients with a stable form and high FeNO compared to an unstable form during exacerbations, where the difference disappeared. This difference supports the usage of FENO in occupational monitoring, in which measurement are generally conducted apart from exacerbations. However, the limitations of this meta-analysis consist in the variety of FeNO measurements’ methods, the lack of data on corticosteroid treatment during FeNO measurement, and the relatively small number of patients included in the studies. Additionally, in COPD patients, smoking is frequent, and smoking significantly reduces the level of FeNO [79].

The utility of FeNO in oCOPD has an epidemiological support, through a prevalence survey conducted on 13,336 subjects, showing an odds ratio (OR) of 1161 in people with FeNO ≤50 ppb exposed to organic dust (organic dust) and a higher OR of 1314 in workers exposed to exhaust fumes. The second study has the limitation of not being based on COPD diagnosis confirmed by a physician.

There are few studies dedicated to a specific occupational exposure potentially generating COPD [80]. Some of them assessed the acute effect of potential COPD agents by measuring FeNO and other markers of inflammation before and after a work shift. Thus, Pelclova D et al. measured FeNO and tumor necrosis factor, leukotriene B4 and E4 in exhaled breath condensate in people exposed to nanoparticles, and Wang et al. measured FeNO and serological markers of inflammation—club cell secretory protein, C-reactive protein, surfactant protein A and interleukin 6—in workers exposed to diesel exhaust fumes. Although the other inflammation biomarkers showed a significant variation, both studies did not find measuring FeNO before and after a work shift useful [81,82]. This may be due to the FeNO variation curve after acute occupational exposure, where a significant increase would occur at 24 h [83]. Moreover, one argument in favor of the persistent response is an inverse relationship between the level of FeNO and the duration of occupational exposure to exhaust diesel.

Another issue related to the clinical application of FeNO is the lack of consensus on the normality of FeNO in COPD. FeNO above 50 ppb is associated with eosinophilic inflammation of the airway but comparing FeNO with sputum eosinophil count does not yet have a clear diagnostic value [77].

Recognition of eosinophilic bronchitis is essential because the absence of nonspecific bronchial hyperreactivity is often an exclusionary element in the diagnosis of asthma. In the particular case of occupational asthma, the lack of bronchial hyperactivity excludes occupational involvement and, implicitly, the secondary prevention measures.

In conclusion, the determination of interleukins in the blood or leukotrienes in exhaled air highlight the type of airway pattern of inflammation [84]. So far, apart from the limited indications of FeNO, these biomarkers have not been translated into clinical practice [81] and should be considered for validation in future studies.

### 3.4. Differentiating between Occupational and Non-Occupational COPD

This is relatively simple if there is only one source of exposure (i.e., smoking or an occupational source), but it is challenging if the person with a documented workplace exposure is also a smoker, which is quite often encountered in clinical practice. Of course, a complete image of the occupational component of COPD implies documenting the exposure. In clinical practice, it is also always easier to identify the occupational exposures when they are ongoing. This becomes challenging to assign the role of risk factors when the exposure has since stopped or if the common symptoms (such as a cough) are considered by the workers as a “normal” way to respond to the contact with a toxicant and not even mentioned to the physician.

Most countries exclude smokers from the category of patients who could claim compensation for oCOPD, because, at individual level, it is difficult to estimate the amount of occupational exposure in the occurrence of disease among smokers. Another reason for the ignorance of the oCOPD is the late onset of the disease, often after retirement or at least after exposure to the noxious occupational agent has stopped.

The prevention interventions must be directed in both smoker and non-smoker populations at risk of oCOPD because smoking association doubles the risk of oCOPD development (from 14% in non-smokers to 31% in smokers) [85]. More intensive smoking cessation programs adapted to the needs of exposed workers are necessary for the overall reduction in the incidence of COPD [86].

### 3.5. Differentiating between COPD and Other Occupational Diseases

This is also challenging because occupational exposure to one single respiratory hazard is very rare. Physical, chemical and biological risk factors may coexist in the same workplace, and their presence has a synergistic effect on lung inflammation development and persistence [87]. For example, in foundries, the exposure to free crystalline silica dust may be associated with the exposure to epoxy resins and/or urea-formaldehyde, gases and metal fumes. Therefore, with long-term occupational exposure, in this sector of activity, the nodular and fibrotic lesions of silicosis may coincide with asthma, oCOPD and lung cancer. Another example is coal miners who are exposed to a mixture of dusts (coal, free crystalline silica, silicates, metals). The oxidative stress induced by inhaled metal particles could be the pathogenic factor of COPD. It has been shown that an elevated blood level of heavy metals (cadmium, lead, mercury and chromium) was negatively associated with both obstructive functional parameters, S-glutathione transferase activity and monocyte glutathione level [88].

From a clinical point of view, chronic cough dominates the clinical picture of various occupational diseases; this can also represent a challenge in diagnosis. It is a symptom frequently encountered in workers exposed to occupational respiratory hazards. Depending on all factors in the work environment and individual reactivity, the occupational diseases can include, apart from oCOPD, occupational asthma, hypersensitivity pneumonitis, hard metal disease, collagenous and noncollagenous pneumoconiosis, beryllium and occupational cancer [89]. A systematic review identified sufficient evidence for both irritant-induced asthma and COPD for exposure to welding fumes, sulfur dioxide, pig farming, or first responders of the World Trade Center (WTC) disaster [90]. An appropriate differential diagnosis work up must be included within the management algorithms of pulmonary occupational diseases because corticosteroid treatment must be avoided especially in the presence of several associated comorbidities. Last but not least, general medical practitioners must improve their adherence to current management guidelines in order to ensure a more accurate and early diagnosis of COPD patients [91,92,93,94,95].

Diagnosing oCOPD, as a result of VGDF exposure, is also challenging. A 12-year cohort study that monitored adult asthmatics found that exposure to VGDF increases the prevalence of the “asthma-COPD overlap”. Respectively, the clinical component of bronchial asthma also associates the predominance of neutrophilic inflammation and other markers of systemic inflammation with a reduction in the degree of obstruction reversibility [96]. In clinical practice, the association of paroxysmal dyspnea, inhalation fever and general manifestations may guide the diagnosis of occupational COPD. Still, most often, clinical evaluation is not sufficient and must be completed with lung function, imaging exam and laboratory tests. For example, the differentiation between asthma, COPD and asthma-COPD overlap encountered in firefighters who cleared debris from WTC relied on measuring eosinophilia and interleukin 4 (IL-4), which increased only in asthma-COPD overlap, and IL-21, which was higher only in isolated asthma and COPD [84].

Differential diagnosis is even more challenging when two or more occupational diseases coexist. As mentioned above, silicosis can coexist with chronic bronchitis due to simultaneous exposure to silica dust and respiratory irritants. However, the relationship is not mandatory: the unique exposure to crystalline silica dioxide, such as in workers drilling in natural stone (“outdoor rock drillers”), can cause only one of the effects, namely the obstructive syndrome, in the absence of nodular lesions of silicosis. In their study from 2020, Ulvestad B et al. underlined the relationship between exposure and bronchial obstruction in non-smokers with longer professional tenure and more intense exposure [97].

Another example is represented by the coexistence of oCOPD with hypersensitivity pneumonitis, for example, found in farmers exposed to organic compounds of plant or animal origin and to insecticides [98,99]. In fact, one of the main comorbidities of hypersensitivity pneumonitis is COPD. For example, in a study conducted in Spain, among patients diagnosed with interstitial lung disease hospitalized between 2014 and 2015, the frequency of COPD among patients with hypersensitivity pneumonitis was 100% [98,99,100,101].

Multiple exposure and comorbidities may highlight other diagnoses in which cough and dyspnea are present. However, the diagnostic concordance between different clinicians is relatively good, if exposure is known [102].

Legislative constraints make the diagnosis of oCOPD challenging to compare its prevalence between countries or between sectors of activity in different countries. For example, a study showed the failure to meet eligibility criteria to the compensation system in Poland (FEV1 reduction > 50% or the lack of sufficient documentation on exposure) hampered the recognition of COPD in almost half of the suspected patients who met the GOLD diagnostic criteria [103]. Other countries are considering COPD as an occupational disease only if a very high level of exposure to occupational hazards is identified. Moreover, the list of etiological agents is very much restricted and many agents are not considered at all for oCOPD recognition and compensation [104].

## 4. Conclusions

The main challenges for oCOPD are the emerging hazards, the lack of early detection and the constraints towards the recognition as occupational disease.

In order to surmount these challenges, all physicians should be aware and report cases in relation to work-related respiratory hazards. They should cooperate with occupational physicians in clarifying the diagnosis and in assuring the proper treatment.

The identification of methods for early detection and monitoring of oCOPD should be a priority for public health research. The implementation of more sensitive methods could allow workplace interventions to discontinue exposure and prevent the evolution to the severe forms of COPD.

## Figures and Tables

**Table 1:** Common workplace hazards associated with occupational COPD.

**1. Exposure to Mineral (Inorganic) Dusts**
Coal
Silica and silicates
Asbestos
Cement
Synthetic fibers
Oil mist
Improper air conditioning systems *
2. Metals **
Cadmium
Osmium
Vanadium
Chlorine
Iron
Titanium
Chromium
Nickel
**3. Organic dust**
Cotton and other textile fibers
Grain
Flour
Wood
Endotoxins
Animal products
Mixture of organic dusts: biomass
**4. Pesticides, insecticides, herbicides**
Heptachlor
Dichlorvos
Permethrin
DDT
Carbofuran
Cyanazine
Paraquat
**5. Gases, vapors and fumes**
Fire smoke
Volatile organic compounds (diesel exhaust ***, paints, varnishes, wax, cleaning or disinfecting products, organic solvents etc.)
Welding ****
Nitrates
Sulphur dioxide
Ozone
Ammonium
Isocyanates

* Improper air conditioning systems contribute to the indoor air pollution by modifying physical factors (temperature, humidity), number of microparticles and concentration in volatile organic compounds, endotoxins and molds in the air. ** Metals are present in workplaces as inorganic dust (e.g., in toner powders) or in the composition of the fumes and gazes (e.g., welding). *** Diesel exhaust contains a large quantity of respirable particles, volatile organic compounds such as benzene, formaldehyde, naphthalene, *n*-hexane, toluene, xylene. **** Welding process creates a mixture of different particles, vapors, fumes and gases (metals, ozone, phosgene, fluorine nitrogen) depending on the type of welding, type of electrode, material to be welded, including the coating of its surface).

## Data Availability

Not applicable.
